# Multi-Information Model for Large-Flowered Chrysanthemum Cultivar Recognition and Classification

**DOI:** 10.3389/fpls.2022.806711

**Published:** 2022-06-06

**Authors:** Jue Wang, Yuankai Tian, Ruisong Zhang, Zhilan Liu, Ye Tian, Silan Dai

**Affiliations:** ^1^Beijing Key Laboratory of Ornamental Plants Germplasm Innovation and Molecular Breeding, Beijing Laboratory of Urban and Rural Ecological Environment, Key Laboratory of Genetics and Breeding in Forest Trees and Ornamental Plants of Ministry of Education, National Engineering Research Center for Floriculture, School of Landscape Architecture, Beijing Forestry University, Beijing, China; ^2^College of Technology, Beijing Forestry University, Beijing, China

**Keywords:** large-flowered chrysanthemum, image classification, cultivar recognition, cultivar classification, deep learning

## Abstract

The traditional Chinese large-flowered chrysanthemum is one of the cultivar groups of chrysanthemum (*Chrysanthemum* × *morifolium* Ramat.) with great morphological variation based on many cultivars. Some experts have established several large-flowered chrysanthemum classification systems by using the method of comparative morphology. However, for many cultivars, accurate recognition and classification are still a problem. Combined with the comparative morphological traits of selected samples, we proposed a multi-information model based on deep learning to recognize and classify large-flowered chrysanthemum. In this study, we collected the images of 213 large-flowered chrysanthemum cultivars in two consecutive years, 2018 and 2019. Based on the 2018 dataset, we constructed a multi-information classification model using non-pre-trained ResNet18 as the backbone network. The model achieves 70.62% top-5 test accuracy for the 2019 dataset. We explored the ability of image features to represent the characteristics of large-flowered chrysanthemum. The affinity propagation (AP) clustering shows that the features are sufficient to discriminate flower colors. The principal component analysis (PCA) shows the petal type has a better interpretation than the flower type. The training sample processing, model training scheme, and learning rate adjustment method affected the convergence and generalization of the model. The non-pre-trained model overcomes the problem of focusing on texture by ignoring colors with the ImageNet pre-trained model. These results lay a foundation for the automated recognition and classification of large-flowered chrysanthemum cultivars based on image classification.

## Introduction

The traditional Chinese large-flowered chrysanthemum (larger-flowered chrysanthemum) is a particular group of chrysanthemum (*Chrysanthemum* × *morifolium* Ramat.) derived from wild Chrysanthemum species through domestication and selection for over 2,600 years in China (Dai et al., 2012). The cultivar group of large-flowered chrysanthemum has over 3,000 cultivars to date, and they exhibit a rich diversity in floral morphology. Thus, this cultivar group possesses excellent aesthetic value and prospects for the market (Zhang et al., 2014a; Dai and Hong, 2017; Su et al., 2019).

Cultivar identification and classification are very important for production and communication (Yu, 1963). Similar to the cultivar classification system for other ornamental plants, such as Lily,^1^ Rosa,^2^ Daffodils,^3^ and Peony,^4^ the classification of large-flowered chrysanthemum is also based on critical morphological traits, such as flower color (Hong et al., 2012), flower type (Zhang et al., 2014b), petal type (Song et al., 2018), and leaf type (Song et al., 2021), and classifies a considerable number of cultivars into multiple groups with high similarity within-group and high variation between groups.

**FIGURE 1:**
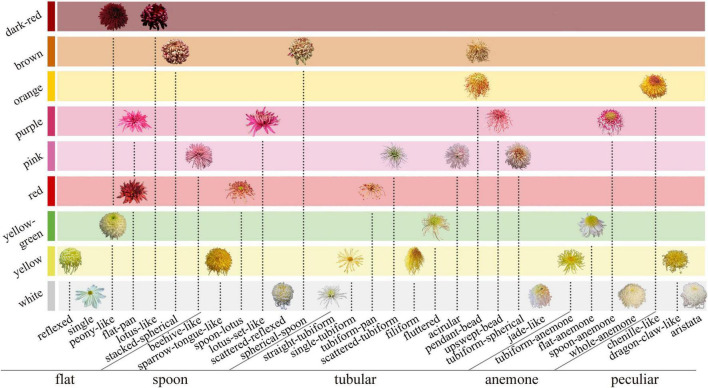
Classification system of large-flowered chrysanthemum based on flower color, petal type, and flower type.

At present, the researchers widely use the classification system of 9-color series based on flower color (Hong et al., 2012), five petal types, and 30 flower types based on flower shape (Wang, 1993). In the former, the large-flowered chrysanthemum is divided into nine color groups by quantitative classification. In the latter, the petal type (flat, spoon, tubular, anemone, and peculiar) is the first criteria of the classification, and the second is the flower shape (the petal details and the combination relationship among petals). The above two systems determine the distribution of floral characteristics in large-flowered chrysanthemum (Figure 1).

However, faced with the vast number of large-flowered chrysanthemum cultivars, morphological variability within a cultivar, and similarity to other cultivars, the above classification system’s efficiency, and accuracy are often challenged.

Deep learning is an emerging area of machine learning for tackling large data analytics problems (Ubbens and Stavness, 2017). As one of the most popular branches of machine learning research, deep learning has been widely employed and has attracted more attention from various domains, such as protein prediction (Le and Huynh, 2019; Tng et al., 2022), plant disease detection (Abade et al., 2021), plant yield, growth prediction (Ni et al., 2020; Shibata et al., 2020), and animal identification (Norouzzadeh et al., 2018; Spiesman et al., 2021).

A significant trend in plant recognition in recent years has been to use deep learning for plant image classification (Wldchen and Mder, 2018). The network that applies to deep learning for plant images classification is the deep convolutional neural network (DCNN), which establishes the classification model by extracting features of plant images. So far, DCNN derived a series of network structures, such as VGG (Visual Geometry Group) (Simonyan and Zisserman, 2015), GoogleNet (Szegedy et al., 2015), and ResNet (Residual Neural Network) (He et al., 2016). ResNet is the first classification network that surpasses human accuracy in classification tasks (Russakovsky et al., 2015). At present, the ResNet-based classification model has been widely used in plant image research, such as plant age judgment (Yue et al., 2021), flowering pattern analysis (Jiang et al., 2020), and root image analysis (Wang et al., 2020).

**FIGURE 2:**
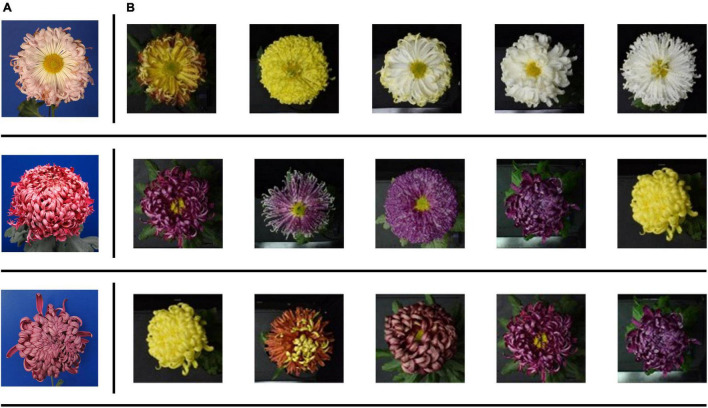
Examples of misclassified images by the pre-training model. **(A)** The test images. **(B)** The Top-5 results.

For plant recognition, users only need to provide images of plant organs, such as leaves (Zhang et al., 2020) and flowers (Seeland et al., 2017; Liu et al., 2019), to complete the recognition of plants. With the application of related research and development, over 30,000 species of plants could be recognized (Mäder et al., 2021). However, because of the difficulties in data acquisition, many deep learning methods used pre-training models based on ImageNet (He et al., 2015, 2016; Russakovsky et al., 2015; Chattopadhay et al., 2018) as the backbone network, but ImageNet-trained CNNs are strongly biased toward recognizing textures and not sensitive to color (Geirhos et al., 2018). When using the classifier constructed by the ImageNet-trained model to test the images of large-flowered chrysanthemum in 2008, we also found that the top-5 results and test images have similar textures but a difference in color (Figure 2).

A number of previous studies utilized deep learning for plant image classification, which only provided single taxonomic information of plants (Waeldchen and Maeder, 2018). A recent study on large-flowered chrysanthemum image classification (Liu et al., 2019) established a recognition model with the output of cultivar name. It cannot fully meet the requirements of large-flowered chrysanthemum recognition and classification. It is important to recognize large-flowered chrysanthemum, and the classification according to corresponding petal type and flower type is also necessary for practical application. These results have practical value in market communication and landscape application.

We also consider the large intra-cultivar visual variation. The large-flowered chrysanthemum belonging to the same cultivars may show considerable differences in their morphological characteristics depending on their different abiotic factors, development stage, and opening periods, which is a challenge to the generalization of the model (Liu et al., 2019).

Based on previous research, for large-flowered chrysanthemum recognition and classification, and to overcome the bias to texture, we proposed a multi-information classification model that can output flower type, petal type, and cultivar name. We also tested the model’s generality on the datasets of different years.

## Materials and Methods

### Plant Material

According to the previous classification system of flower type and color (Wang, 1993; Hong et al., 2012), we selected large-flowered chrysanthemums in the chrysanthemum resource nursery (in Dadongliu nursery in Beijing) of the research group. To cover all flower colors, petal types, and flower types of chrysanthemum cultivars, we selected 126 cultivars in 2018 (as shown in Supplementary Table 1) and 117 cultivars in 2019 (as shown in Supplementary Table 2). After removing the duplication, there were 213 cultivars in 2018 and 2019.

Referring to the Chinese Chrysanthemum book (Zhang and Dai, 2013), we accomplished the cultivation and management of large-flowered chrysanthemum in the Dadongliu nursery in Beijing in 2018 and 2019, respectively.

### Image Acquisition and Labeling

The image acquisition of large-flowered chrysanthemum was carried out during flowering periods in November to December in 2018 and 2019. The image acquisition device and image acquisition process are the same as the study by Liu et al. (2019). The image resolution was 6,000 × 6,000 pixels, and the format was PNG. In the gathered images, each cultivar had at least 2–3 individuals. We photographed each individual from the top view and oblique views while ignoring the background.

All the collected images were accurately and uniformly marked using LabelImg v1.7.0 software. See Supplementary Table 3 for cultivar name, petal type, and flower type marking.

### Dataset Construction

#### 2018 Dataset (Training Dataset and Validation Dataset)

**FIGURE 3:**
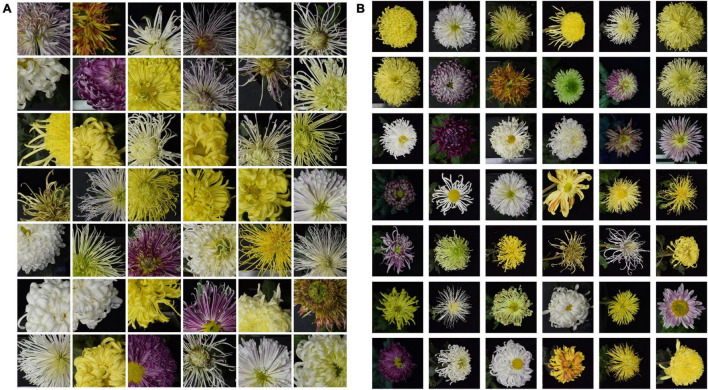
Sample images from dataset training and validation. **(A,B)** Are training and validation dataset, respectively.

The 2018 dataset contained 126 cultivars. To balance the samples, we randomly selected 80 images from each cultivar for 10,080 images. A total of 80% of the images were used for training and 20% for validating. Data augmentation plays a crucial role in improving classification performances. However, large-flowered chrysanthemum recognition is similar to face recognition, so some common data enhancement methods are not adopted. For example, color is essential information for flowers. Therefore, methods such as color jitter and gray scales are unsuitable for actually identified scenes. In addition, the symmetry of chrysanthemum structure, rotation, and flip operations are invalid. For large-flowered chrysanthemum image recognition, the deep network model focuses on the local information of the image (Liu et al., 2019), so the cropping is only for the training dataset. The original image of the training dataset is scaled to 256 × 256 pixels then randomly cropped to 224 × 224 pixels image patch. By random cropping, we expanded the number of the training dataset (Figure 3A) by 10 times to 80,640. The validation dataset (Figure 3B) is 2,016 images used to determine model architecture and hyper-parameters.

#### 2019 Dataset (Test Dataset)

**FIGURE 4:**
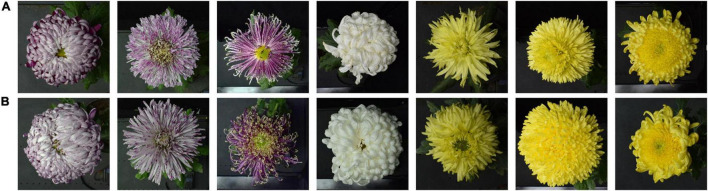
Some sample images in the 2018 and 2019 datasets. Row **(A,B)** belong to the 2018 and 2019 datasets, respectively.

The 2019 dataset contained 2,556 images belonging to 117 cultivars, including 640 images of 30 similar cultivars as the 2018 dataset. This dataset formed the test dataset. Figure 4 shows some of the same cultivars in 2018 and 2019. Because of the differences in climate environment and photo time in 2018 and 2019, the flower type of the same cultivar has changed to some extent, which can test the model’s generalization.

### Devices

The models were built and trained on the Ubuntu 16.04 system, based on Intel Xeon Gold 5120 CPU and 4 NVIDIA Titan Xp 16GB GPU hardware platform.

### Approach

**FIGURE 5:**
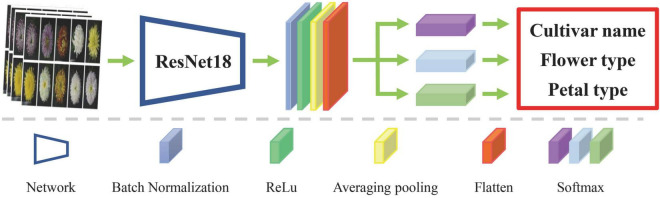
Architecture of the network model.

Many researchers used the pre-trained network model on the ImageNet dataset to extract image features. As mentioned above, ImageNet-trained CNNs are strongly biased toward recognizing textures and ignoring color. However, for large-flowered chrysanthemum, color is an essential characteristic for classification. We used the script of non-pre-trained ResNet18 (He et al., 2016) as the backbone network. Due to the limited amount of data, we abandoned the deeper network, such as ResNet50. The network comprised three parallel softmax classifiers to get a richer feature representation of large-flowered chrysanthemum images. It means that the network model has three outputs about botanical information of cultivar name, flower type, and petal type, respectively. Figure 5 shows the network structure. The features of the images are flatten-layer output with 512-dimensions. Keras was used for our experiments. The DCNN was initialized by He initialization (He et al., 2015).

### Model Training

The total loss function (1) includes cultivar name loss, flower type loss, and petal type loss.


(1) $$\text{Loss}={\text{Loss}}_{\mathrm{name}\,\_\,\mathrm{cultivar}}+{\text{Loss}}_{\mathrm{types}\,\_\,\mathrm{flower}}+{\text{Loss}}_{\mathrm{types}\,\text{\_}\,\mathrm{petal}}$$

The label smoothing (Szegedy et al., 2016) was used to increase the convergence rate in the training phase. The optimization method was the Stochastic Gradient Descent (SGD), Momentum of 0.9, training used a batch size of 32, and was terminated after 40 epochs.

In training, the network’s weights are updated according to a certain strategy. The weight update function is defined as (2).


(2) W+=α×gradient

α is the learning rate, the gradient is the corresponding weight gradient.

Because the model used the script of the ResNet18 network with nearly 32M parameters and 80,640 pictures in the training dataset, the network was easy to overfit. To avoid overfitting, we used different learning rate adjustment methods (Rosebrock, 2021) for comparison.

In the standard-decay strategy, the initial init_lr = 1e-2, the function is (3).


(3) $$\alpha =\text{init}\,\_\,\text{lr}\times \frac{1.0}{1.0+\text{decay}\times \text{iterations}},\text{decay}=\frac{\text{init}\,\_\,\text{lr}}{\text{echos}}$$

In the step-decay strategy, the initial init_lr = 1e-2, the formula is (4).


(4) $$\alpha_{E+1}=\alpha_{I}\times {\text{F}}^{\left(1+E\right)/D}$$

*α_*I*_* represents the initial learning rate, *F* represents the learning rate factor, *F* = 0.25, *E* represents the current *echo*, *D* represents each *echo* to adjust the learning rate, *D* = 10.

In the line-decay strategy, the initial *init_lr* = 1e-2, the formula is (5).


(5) $$\alpha_{E+1}=\alpha_{I}\times {\left(1-\frac{\text{E}}{\text{echos}}\right)}^{\mathrm{pow}}$$

When *pow* = 1, it means the line-decay approach.

In the poly-decay strategy, the initial init_lr = 1e-2 (when pow = 5, it means the poly-decay strategy), the formula is (5).

### Evaluation of Results

We used the Top-k accuracy to evaluate the model. If the K results include the correct categories, we consider the results valid. It took the average value of all images in each cultivar test dataset as the Top-1 and Top-5 accuracy. In addition, we used the F1-score and recall as the evaluation metrics.

### Feature Analysis

After training, we extracted the 512-dimensional image features for the 126 cultivars in the 2018 validation dataset and 87 cultivars in the 2019 test dataset. By the AP clustering (Frey and Dueck, 2007), the correlation between image features and large-flowered chrysanthemum phenotype was analyzed. To explore the class separability of cultivars in the PCA space, we colored the points according to their characteristic labels.

## Results

### Model Training

**FIGURE 6:**
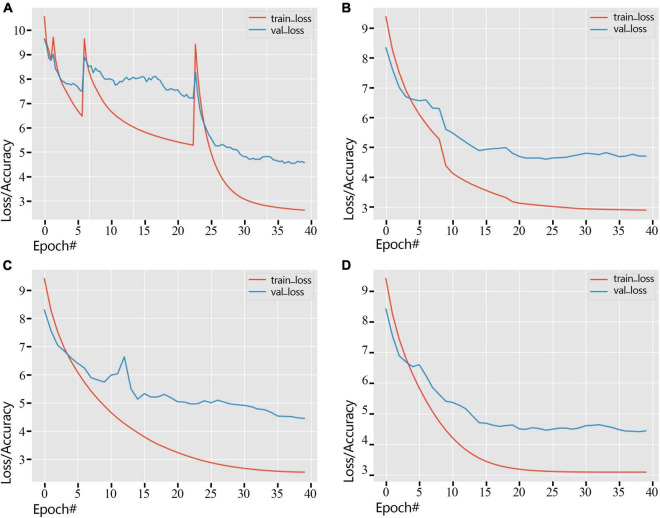
Error variation of training dataset and validation dataset under different strategies (2018 dataset). **(A)** Standard-decay. **(B)** Step-decay. **(C)** Line-decay. **(D)** Poly-decay.

We showed the loss change process of the training dataset and the validation dataset by different learning rate adjustment methods in Figure 6. As shown in Figure 6A, Standard-decay caused the convergence to be unstable, causing the loss curve to spike twice. At the same time, other learning rate adjustment methods were relatively stable, and the loss curve maintained stability. In the training process, the loss of the validation dataset gradually decreases, and the loss of the training process using the poly-decay strategy decreases most smoothly (Figure 6D), which reflects that the poly-decay strategy can ensure the convergence of the network.

**FIGURE 7:**
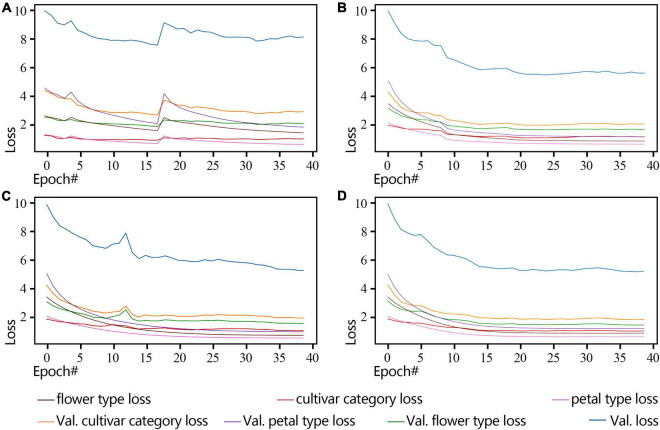
ResNet18 Loss curve of a non-pre-training model during training and validation (2018 dataset). **(A)** Standard-decay. **(B)** Step-decay. **(C)** Line-decay. **(D)** Poly-decay.

By observing the loss changes of the three classifiers, i.e., cultivar name, flower type, and petal type, we obtain the best convergence result using the poly-decay strategy (Figure 7). Among the three classifiers, the petal type classifier has the highest accuracy because the model pays more attention to local features such as petal type than global features such as cultivar name and flower type (Figure 7D).

The validation accuracies of Top-1 and Top-5 of cultivar name, flower type, and petal type of 2018 validation dataset using four kinds of decay strategies are shown in Table 1. Except that the petal type of Top-1 accuracy is lower than the step-decay strategy, the other accuracies of the poly-decay are the highest, poly-decay also had the highest recall and F1-score.

**TABLE 1:** Comparison of decay strategy performance in the validation dataset.

Decay strategy	Cultivar name classification				Petal type classification				Flower type classification			
	Top-1 (%)	Top-5 (%)	recall	F1-score	Top-1 (%)	Top-5 (%)	recall	F1-score	Top-1 (%)	Top-5 (%)	recall	F1-score
Standard-decay	73.96	94.98	0.69	0.68	78.27	98.34	0.78	0.77	72.68	95.08	0.71	0.7
Step-decay	74.15	95.45	0.72	0.71	**82.43**	99.29	**0.82**	0.81	72.92	96.69	0.71	0.7
Line-decay	76.04	96.54	0.74	0.73	81.49	99.34	0.8	0.79	74.91	97.3	0.73	0.72
Poly-decay	**77.61**	**97.16**	**0.78**	**0.77**	81.68	**99.72**	0.8	**0.79**	**76.42**	**98.44**	**0.75.**	**0.74**

*Text in bold indicates the best value in each category.*

### Model Generalization Performance

The generalization ability to the 640 images of the same cultivars in the 2019 dataset and the 2018 dataset, while comparing the influence of image cropping, is shown in Table 2. For petal type, a local feature, the model’s accuracy with cropping image patches is higher than that without cropping. Compared with the petal type, the flower type, which reflects the overall features of the large-flowered chrysanthemum, the model’s accuracy with cropping image patches is lower than that without cropping. For cultivar name, the model’s accuracy with cropping image patches is four times higher than without cropping, confirming the previous conclusion that the large-flowered chrysanthemum classification model focuses on the local (Liu et al., 2019).

**TABLE 2:** Influence of cropping or non-cropping models on a generalization of 2019 cultivars classification.

	Cultivar name classification accuracy (%)		Flower type classification accuracy (%)		Petal type classification accuracy (%)	
	Top-1	Top-5	Top-1	Top-5	Top-1	Top-5
Non-cropping	8.15	25.00	75.64*	95.4*	61.5	88.37
Cropping	32.81*	70.62*	61.25	84.37	70.12*	96.34*

The superscript “*” denotes a significant difference (P < 0.05) between cropping or non-cropping using one-way ANOVA. The highest accuracy is marked in bold face.

**FIGURE 8:**
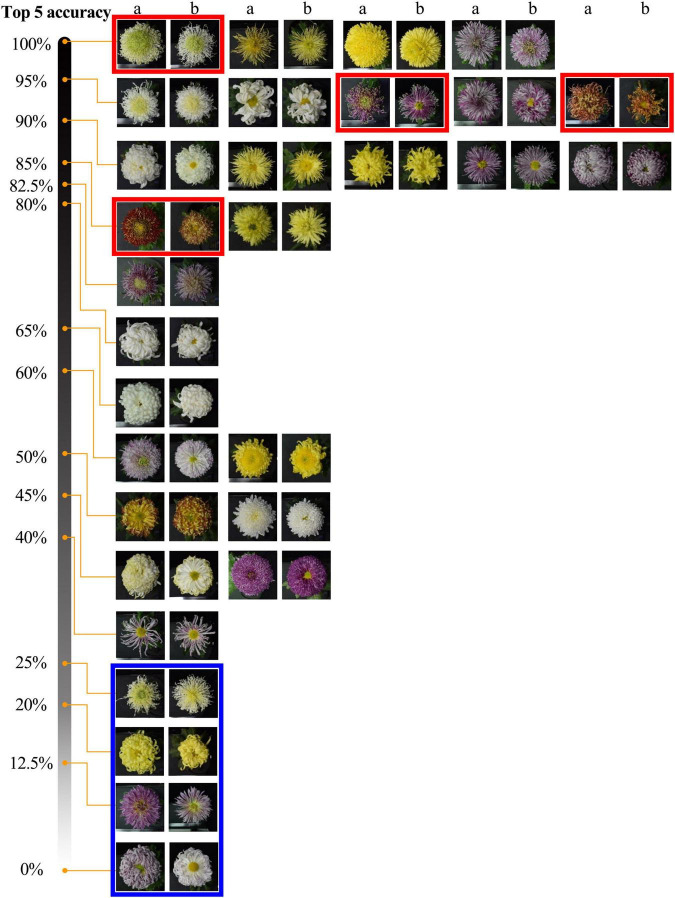
Top-5 recognition results of 2019 dataset. The two adjacent columns are the same cultivar, a and b belong to the 2019 and 2018 datasets. Red and blue frames indicate some cultivars with noticeable morphological changes but with high and low identification accuracy.

The model could accurately identify the morphological changes of the same cultivars to some extent (Figure 8). The results show that among 30 cultivars, the accuracy of 9 cultivars is greater than or equal to 90%, and 18 cultivars are greater than or equal to 80%. For some cultivars (red frames marked) with significant morphological changes, the model can identify them with a high accuracy rate (over 85%). We also found some cultivars (blue frames marked) that changed to the degree that exceeded the model’s generalization, and we will further analyze this problem in our discussion.

**FIGURE 9:**
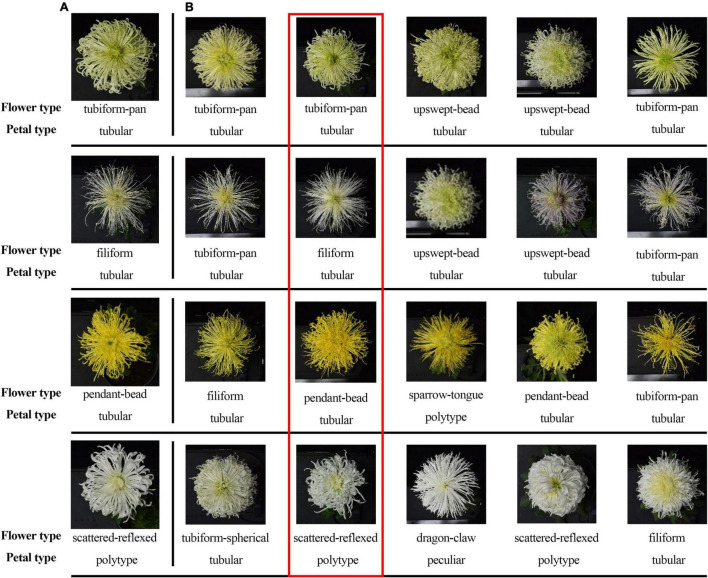
Top-5 results of some cultivars. **(A)** is test images, **(B)** is Top-5 results, and the possibility from left to right gradually reduced. The text below the picture means the corresponding flower type and petal type for each cultivar.

The Top-5 results’ details of some cultivars are shown in Figure 9. The Top-5 results are consistent in the flower color, which indicates that the model can recognize the vital character of flower color. When focusing on petal type and flower type, we found the model has higher accuracy for petal type.

### Affinity Propagation Cluster Analysis

**FIGURE 10:**
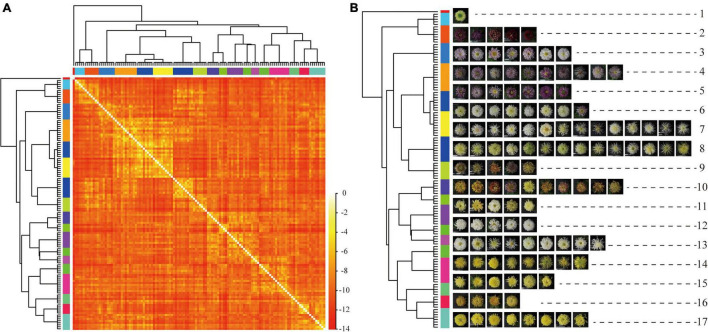
AP clustering analysis of image features of 126 cultivars. **(A)** AP clustering heat map of image features of 126 cultivars. **(B)** AP clustering analysis of image features of 126 cultivars. Note: each row represents a cluster, and the red box is the central cultivar of each category.

The AP clustering algorithm was used to cluster and compare the image features of the 2018 validation dataset (Figure 10). The maximum number of iterations is 1,000, the attenuation coefficient is λ = 0.9, and the convergence condition is δ = 100. The Ap clustering result is shown in Figure 10B. The 126 cultivars in 2018 were automatically clustered into 17 categories and the central cultivars of each category were extracted, as shown in the red frame.

The large-flowered chrysanthemum images were not labeled with any color information, and the AP clustering still showed prominent clustering features by color. The second cluster belonged to dark red, the 12th cluster belonged to white, and the 14th, 15th, and 17th clusters belonged to yellow. While in other clusters, although they were not all the same color, the cultivars in the same clusters belonged to similar colors, such as the third, fourth, and fifth clusters belonged to pink-purple; sixth and seventh clusters are yellow-white; 14th, 15th, 16th, and 17th are yellow-orange.

For the petal type features, the image features of tubular petals were the most discriminative; most were clustered in clusters seventh and eighth, while the flat petal, the spoon petal, and the peculiar petal had poor clustering distribution. The spoon petal was the most relaxed.

### Principal Components Analysis

Based on the non-pre-training Resnet18, we extracted the image feature of 87 new cultivars from the test dataset in 2019 for PCA. We show the results in Table 3. The interpretation degree of the first principal component (PC1) to the original data is 11.22%, and PC2 to the original data is 9.67%. The first two principal components (PCs) accounted for 20.89% of the total variance, while the first 38 PCs account for approximately 90% of the total variance in the original data.

**TABLE 3:** Interpretation of principal component analysis.

Principal components	Eigenvalues	Importance of components	Cumulative proportion
PC1	57.437	11.218	11.218
PC2	49.524	9.673	20.891
PC38	1.707	0.333	90.286
PC185	0.050	0.010	99.003
PC492	0.003	0.001	99.990

**FIGURE 11:**
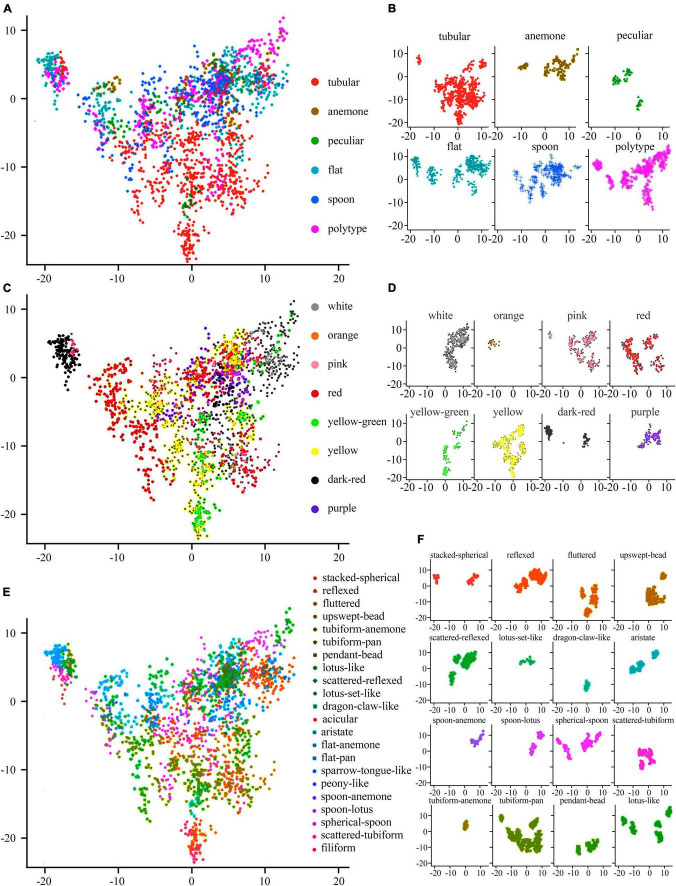
Distribution of various flower colors, petal types, and flower types on the first two PCs. **(A,C,E)** The comprehensive distribution results of flower colors, petal types, and flower types, respectively. **(B,D,F)** The individual distribution results of flower colors, petal types, and flower types, respectively.

The 1,916 image features were visualized on the first two PCs, and colored by flower color (Figures 11C,D), petal type (Figures 11A,B), and flower type (Figures 11E,F), respectively. The image features of white, orange, purple, yellow, and pink large-flowered chrysanthemum cultivars were concentrated. The image features of yellow-green, red, and dark-red large-flowered chrysanthemum cultivars were scattered.

For the distribution of petal types, we can clearly distinguish the tubular from other petal types on the distribution map (Figure 11A), while other petal types were mixed above the distribution map.

For the flower types (Figures 11E,F), it shows that the first two PCs have a certain explanatory effect on the image characteristic flower types of large-flowered chrysanthemum cultivars. Among flower types, Lotus set like, dragon claw like, and tubiform anemone have the best aggregation. However, some flower types, such as tubiform pan, Lotus like, and spherical spoon, not only have poor aggregation but also have a great overlap with each other.

## Discussion

### Dynamic Identification of Large-Flowered Chrysanthemum Image

**FIGURE 12:**
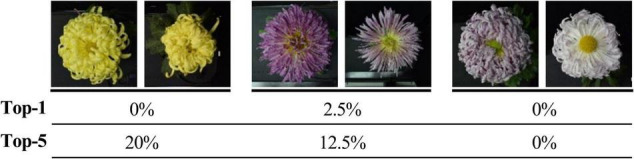
Top-1 and Top-5 identification accuracy of some cultivars. Two adjacent images are the same cultivars from datasets in 2018 and 2019, respectively.

For the 30 same cultivars in 2018 and 2019, the results of the Top-1 and Top-5 were only 32.81% and less than 70%. It was apparent that the vast differences in the images of large-flowered chrysanthemum obtained at different flowering stages directly affected the results of recognition and classification. For some cultivars with low recognition rates, the images obtained in different years have apparent flower color and flower type changes. Sometimes this change shows a comprehensive and complex shift in floral characteristics, but the model lacks sufficient generalization for this circumstance (Figure 12).

The large-flowered chrysanthemum belonging to the same cultivars may show considerable changes in different developmental stages, nutrient levels, or stress conditions. The single classification network cannot be accurate enough for this dynamic process. In terms of dynamic phenotypic identification, good progress has been made in related studies on leaves. By obtaining leaf images at different growth stages (Zhang et al., 2021) or under different stress conditions (Hao et al., 2020), researchers have established a classification model that can recognize the changing leaves through multi-feature or multi-scale input. However, compared to leaf images, flowers are only available during a short period of the year. Due to being complex 3D objects, there is a considerable number of variations in viewpoint, occlusions, and scale of flower images. In the future, one problem to be solved is establishing a dynamic identification model that can accurately identify the 2D images of large-flowered chrysanthemum in various states.

### Deep Features Analysis

It is difficult to interpret the principle of deep network decision-making and analyze the deep features (Castelvecchi, 2016; Lipton, 2018). When the primary purpose is to advance biological research based on accurate prediction, the interpretability of the deep learning model becomes crucial. Whether the classification model of large-flowered chrysanthemum has botanical application value, it needs to analyze whether the classification model is based on the relevant botanical characters and quantify botanical characters’ importance. Liu trained the VGG-16 network with the transfer learning method and extracted 4096-dimensional features, but the clustering results and visual analysis of extracted deep features did not reflect the general distribution rule (Liu et al., 2019). In this paper, the AP clustering and PCA analyzed the 512-dimensional features clustering, and the AP clustering showed that flower color presents high aggregation. For PCA, PC1, and PC2 only account 20.89% of the original data. The image features of chrysanthemum cultivars of each flower color, petal type, and flower type overlap in the first two PCs, and the boundary between different groups is not obvious. It shows that PC1 and PC2 are still not enough to explain the petal type, flower type, and flower color. Further research still needs more principal components.

## Conclusion

The results show that the Top-5 accuracy of the ResNet18 non-pre-training model based on the poly-decay strategy is 70.62%. The image processing, model training scheme, and learning rate adjustment method significantly influence the model’s generalization performance. The AP clustering was used to analyze the deep features. The AP clustering result showed that the 126 cultivars in the 2018 dataset were divided into 17 clusters; the flower color and the petal type clustering effect were better than the flower type. Because the structure of the classification network limits the number of categories, it cannot meet the requirement of category increase. In this case, metric learning is a solution.

## Data Availability Statement

The raw data supporting the conclusions of this article will be made available by the authors, without undue reservation.

## Author Contributions

YT conceived the study. SD completed the work of cultivars’ confirmation, guided sample selection, and cultivation. JW and ZL undertook the cultivation, sample collection, and data labeling of the chrysanthemum. YT and RZ performed the experiments, analyzed the data, and prepared the figures and tables. JW and YKT completed the first draft. SD and YT revised the manuscript. All authors read and approved the final manuscript.

## Conflict of Interest

The authors declare that the research was conducted in the absence of any commercial or financial relationships that could be construed as a potential conflict of interest.

## Publisher’s Note

All claims expressed in this article are solely those of the authors and do not necessarily represent those of their affiliated organizations, or those of the publisher, the editors and the reviewers. Any product that may be evaluated in this article, or claim that may be made by its manufacturer, is not guaranteed or endorsed by the publisher.

## Footnotes

1
https://www.lilies.org/culture/types-of-lilies

2
https://www.rose.org/single-post/rose-classifications

3
https://thedaffodilsociety.com/a-guide-to-dafodils/classification-system

4
https://americanpeonysociety.org/learn/herbaceous-peonies/flower-types-anatomy
