# Supplementary material for: Multi-Information Model for Large-Flowered Chrysanthemum Cultivar Recognition and Classification
Source: Front Plant Sci. 2022 Jun 6;13:806711. doi: 10.3389/fpls.2022.806711 (PMC9208330; doi:10.3389/fpls.2022.806711)
Supplement: Supplementary file 1 [file Table_1.DOCX]

Supplementary Material

# Supplementary Table Ⅰ

| **Table S1.** Large-flowered chrysanthemum cultivars information and AP cluster results in 2018. | | | | | |
| --- | --- | --- | --- | --- | --- |
| cultivar number | cultivar name | AP cluster | Flower type | Petal type | Flower color |
| 489 | Lvyishizhe | 1 | tubiform-pan | tubular | yellow-green |
| 39 | Mohe | 2 | flat-pan | flat | dark-red |
| 216 | Tianxiayipin | 2 | chenille-like | peculiar | dark-red |
| 434 | Xuezhaohongmei | 2 | lotus-like | flat | red |
| 634 | Lichun | 2 | lotus-set-like | spoon | red |
| 998 | Gangtiezhanjiang | 2 | stacked-spherical | flat | dark-red |
| 40 | Qingshuihehua | 3 | spoon-lotus | spoon | pink |
| 416 | Gushuifengui | 3 | flat-anemone | anemone | purple |
| 493 | Sashuangyingzi | 3 | sparrow-tongue-like | spoon | pink |
| 505 | Fuguifengliu | 3 | peony-like | flat | purple |
| 616 | Fenhehua | 3 | lotus-like | flat | pink |
| 687 | Chunshuangdouxue | 3 | aristata | peculiar | pink |
| 750 | Posuonufang | 3 | tubiform-pan | tubular | pink |
| 30 | Xiuhuapo | 4 | beehive-like | polytype | red |
| 88 | Yuniangsuoyi | 4 | upswept-bead | tubular | pink |
| 145 | Banzhongyuhu | 4 | fluttered | polytype | white |
| 276 | Yanzhihe | 4 | lotus-like | flat | white |
| 307 | Xiangshanchufeng | 4 | filiform | tubular | purple |
| 381 | Fenxianmingzhu | 4 | upswept-bead | tubular | red |
| 547 | Chaoqunshang | 4 | tubiform-pan | tubular | dark-red |
| 662 | Fenyusongzhen | 4 | tubiform-pan | tubular | pink |
| 811 | Xishitu | 4 | upswept-bead | tubular | pink |
| 849 | Zixianchuanzhen | 4 | tubiform-pan | tubular | purple |
| 8 | Diebaochunfeng | 5 | stacked-spherical | polytype | pink |
| 160 | Fenweixian | 5 | aristata | peculiar | pink |
| 248 | Ziruyi | 5 | lotus-like | flat | purple |
| 329 | Ziyanfanfei | 5 | chenille-like | peculiar | purple |
| 350 | Zilongxianzhao | 5 | dragon-claw-like | peculiar | purple |
| 480 | Zilouzi | 5 | tubiform-pan | tubular | purple |
| 719 | Yuhuchunse | 5 | reflexed | flat | pink |
| 3 | Yulongnaohai | 6 | scattered-reflexed | polytype | white |
| 16 | Bohexiang | 6 | scattered-reflexed | polytype | white |
| 27 | Zidiefanfei | 6 | scattered-reflexed | polytype | purple |
| 41 | Yudiechi | 6 | reflexed | flat | white |
| 76 | Ruixueqinian | 6 | stacked-spherical | flat | white |
| 164 | Renmiantaohua | 6 | stacked-spherical | flat | pink |
| 192 | Wusefurong | 6 | spherical-spoon | spoon | pink |
| 200 | Baiguanqiu | 6 | tubiform-spherical | tubular | white |
| 32 | Yulouchun | 7 | lotus-set-like | polytype | pink |
| 42 | Yulingguan | 7 | straight-tubiform | tubular | white |
| 144 | Qianchifeiliu | 7 | scattered-tubiform | tubular | white |
| 158 | Qianshouguanyin | 7 | dragon-claw-like | peculiar | white |
| 226 | Fengshangshuipei | 7 | tubiform-pan | tubular | white |
| 394 | Tingtingyuli | 7 | tubiform-pan | tubular | white |
| 396 | Yemafenzong | 7 | pendant-bead | tubular | white |
| 412 | Hewuxiangyun | 7 | tubiform-pan | tubular | pink |
| 423 | Zhangmule | 7 | scattered-tubiform | tubular | pink |
| 560 | Sanjiangfeiyan | 7 | filiform | tubular | white |
| 625 | Botuquan | 7 | upswept-bead | tubular | white |
| 720 | Yudaxichuang | 7 | tubiform-pan | tubular | white |
| 731 | Juedaifengzi | 7 | tubiform-pan | tubular | pink |
| 816 | Xichuangxiyu | 7 | filiform | tubular | red |
| 53 | Chunshuilvbo | 8 | tubiform-pan | tubular | yellow-green |
| 74 | Yulourenzui | 8 | upswept-bead | tubular | white |
| 167 | Bihaiyinlong | 8 | spoon-lotus | spoon | yellow |
| 178 | Lvsongzhen | 8 | acirular | tubular | yellow-green |
| 179 | Doulvyishang | 8 | tubiform-pan | tubular | yellow-green |
| 184 | Lvshuizhangliu | 8 | tubiform-pan | tubular | yellow-green |
| 258 | Fangxiqiuyu | 8 | upswept-bead | tubular | yellow-green |
| 389 | Baiyuzhulian | 8 | pendant-bead | tubular | white |
| 552 | Laorenfa | 8 | tubiform-pan | tubular | white |
| 592 | Yinfaxiunv | 8 | tubiform-spherical | polytype | white |
| 656 | Wuhemang | 8 | fluttered | tubular | white |
| 663 | Tangyuluhui | 8 | fluttered | tubular | yellow |
| 716 | Nijinhongguan | 8 | tubiform-pan | tubular | yellow |
| 1022 | Lanhuifengzi | 8 | tubiform-pan | tubular | white |
| 138 | Saijinhua | 9 | tubiform-pan | tubular | yellow |
| 189 | Zhubidianchun | 9 | lotus-set-like | polytype | red |
| 325 | Fenghuangyi | 9 | fluttered | tubular | orange |
| 606 | Fuguichunrong | 9 | scattered-reflexed | spoon | red |
| 734 | Rongchengguyin | 9 | tubiform-pan | tubular | dark-red |
| 37 | Xuri | 10 | flat-pan | flat | yellow |
| 58 | Jinbeidahong | 10 | peony-like | flat | red |
| 104 | Ziruigong | 10 | flat-anemone | polytype | red |
| 185 | Lvping | 10 | lotus-like | polytype | yellow |
| 224 | Cailongzhao | 10 | dragon-claw-like | peculiar | yellow |
| 272 | Zhuzijiaolong | 10 | dragon-claw-like | peculiar | red |
| 290 | Nijinqiehua | 10 | beehive-like | spoon | red |
| 343 | Jiangfengyuhuo | 10 | flat-pan | flat | pink |
| 487 | Jinbianhong | 10 | lotus-like | flat | red |
| 845 | Hupoqiu | 10 | stacked-spherical | flat | orange |
| 123 | Hongshiba | 11 | single | flat | red |
| 129 | Dafengge | 11 | scattered-reflexed | spoon | white |
| 279 | Nenzhuyusun | 11 | scattered-reflexed | spoon | yellow |
| 461 | Tangyuyurong | 11 | pendant-bead | tubular | yellow |
| 646 | Kunlunjixue | 11 | stacked-spherical | polytype | white |
| 170 | Baixuegongzhu | 12 | scattered-reflexed | spoon | white |
| 172 | Xuetao | 12 | stacked-spherical | flat | white |
| 486 | Yushizidai | 12 | single | flat | white |
| 586 | Bingxueshijie | 12 | spherical-spoon | polytype | white |
| 721 | Bingqingyujie | 12 | stacked-spherical | flat | white |
| 34 | Luhuayueying | 13 | tubiform-pan | polytype | white |
| 50 | Tianewu | 13 | scattered-reflexed | polytype | white |
| 159 | Baiweixian | 13 | aristata | peculiar | white |
| 303 | Libaibei | 13 | lotus-like | flat | white |
| 340 | Shanwuyinshe | 13 | scattered-reflexed | spoon | white |
| 345 | Yinlianhehua | 13 | lotus-like | flat | white |
| 437 | Fengxuezhulou | 13 | fluttered | tubular | white |
| 672 | Muronghanxiao | 13 | straight-tubiform | tubular | white |
| 768 | Yuhuanpiaowu | 13 | flat-pan | polytype | white |
| 95 | Jinxianchuizhu | 14 | pendant-bead | tubular | yellow |
| 121 | Huangxiangli | 14 | straight-tubiform | tubular | yellow |
| 125 | Yuemingxingxi | 14 | tubiform-anemone | polytype | yellow |
| 151 | Jinsihou | 14 | filiform | tubular | yellow |
| 173 | Huanghexianzhu | 14 | pendant-bead | tubular | yellow |
| 249 | Xiaonianjinmei | 14 | sparrow-tongue-like | polytype | yellow |
| 419 | Lvkongque | 14 | upswept-bead | tubular | yellow-green |
| 848 | Hefengshuyu | 14 | tubiform-pan | tubular | yellow |
| 207 | Ruizhugong | 15 | tubiform-anemone | anemone | yellow |
| 231 | Huangguanqiu | 15 | tubiform-spherical | polytype | yellow |
| 514 | Nijinhudie | 15 | sparrow-tongue-like | spoon | dark-red |
| 603 | Dapengzhanchi | 15 | spoon-lotus | spoon | yellow |
| 638 | Jinganghuiyue | 15 | jade-like | tubular | yellow |
| 850 | Jinfengdie | 15 | scattered-reflexed | polytype | yellow |
| 174 | Honghuqiu | 16 | stacked-spherical | flat | orange |
| 314 | Jinluanzhiguang | 16 | reflexed | polytype | white |
| 633 | Gufomian | 16 | spherical-spoon | spoon | yellow |
| 812 | Nijinqiu | 16 | stacked-spherical | flat | yellow |
| 193 | Huangmaoju | 17 | aristata | peculiar | yellow |
| 234 | Yuezhonggui | 17 | spoon-anemone | anemone | yellow |
| 365 | Fenhuangxiaoyue | 17 | reflexed | flat | yellow |
| 440 | Jinxie | 17 | lotus-like | polytype | yellow |
| 458 | Qiehuahuang | 17 | tubiform-pan | tubular | yellow |
| 506 | Shierjinchai | 17 | scattered-reflexed | spoon | yellow |
| 655 | Xiangrikui | 17 | reflexed | polytype | yellow |
| 804 | Jinsihuang | 17 | upswept-bead | tubular | yellow |

# Supplementary Table Ⅱ

| **Table S2.** Large-flowered chrysanthemum cultivars information in 2019. | | | | |
| --- | --- | --- | --- | --- |
| cultivar number | cultivar name |  |  |  |
| 8 | Diebaochunfeng |  |  |  |
| 14 | Nijinbao |  |  |  |
| 30 | Xiuhuapo |  |  |  |
| 44 | Nanchaofendai |  |  |  |
| 45 | Yulongxu |  |  |  |
| 63 | Huanghejuanlang |  |  |  |
| 79 | Jinzhangchenglu |  |  |  |
| 85 | Wenjingwuwei |  |  |  |
| 122 | Lvyihongshang |  |  |  |
| 139 | Taoshanjunxiu |  |  |  |
| 155 | Sheyingqiangchu |  |  |  |
| 160 | Fenweixian |  |  |  |
| 168 | Jinxiaguan |  |  |  |
| 169 | Zheshimao |  |  |  |
| 172 | Xuetao |  |  |  |
| 175 | Fensongzhen |  |  |  |
| 185 | Lvping |  |  |  |
| 207 | Ruizhugong |  |  |  |
| 230 | Taiyechihe |  |  |  |
| 231 | Huangguanqiu |  |  |  |
| 234 | Yuezhonggui |  |  |  |
| 237 | Guandongdaxia |  |  |  |
| 238 | Bailuhengjiang |  |  |  |
| 255 | Yushimengxing |  |  |  |
| 258 | Fangxiqiuyu |  |  |  |
| 263 | Huanshuijingui |  |  |  |
| 269 | Zhushahongshuang |  |  |  |
| 279 | Nenzhuyusun |  |  |  |
| 282 | Fenxuanqiu |  |  |  |
| 290 | Nijinqiehua |  |  |  |
| 299 | Luoshen |  |  |  |
| 303 | Libaibei |  |  |  |
| 307 | Xiangshanchufeng |  |  |  |
| 325 | Fenghuangyi |  |  |  |
| 326 | Laosengyi |  |  |  |
| 329 | Ziyanfanfei |  |  |  |
| 330 | Panlongchunxiao |  |  |  |
| 332 | Nijinbaojie |  |  |  |
| 350 | Zilongxianzhao |  |  |  |
| 351 | Xiaomoqiu |  |  |  |
| 353 | Moxie |  |  |  |
| 356 | Baidouqiu |  |  |  |
| 381 | Fenxianmingzhu |  |  |  |
| 386 | Yinpantuogui |  |  |  |
| 390 | Qilinjiao |  |  |  |
| 408 | Jinxingliuxia |  |  |  |
| 439 | Baixueta |  |  |  |
| 440 | Jinxie |  |  |  |
| 450 | Yulongxu |  |  |  |
| 459 | Jindingdiaoyuan |  |  |  |
| 482 | Peichengzhiqiu |  |  |  |
| 485 | Jinmajiongfeng |  |  |  |
| 486 | Yushizidai |  |  |  |
| 487 | Jinbianhong |  |  |  |
| 493 | Sashuangyingzi |  |  |  |
| 494 | Annigongzhu |  |  |  |
| 504 | Tangyufenfeiwu |  |  |  |
| 553 | Yangchunbaixue |  |  |  |
| 560 | Sanjiangfeiyan |  |  |  |
| 567 | Xiahui |  |  |  |
| 569 | Hongtuogui |  |  |  |
| 586 | Bingxueshijie |  |  |  |
| 601 | Bingfengxuelian |  |  |  |
| 617 | Wanyuelianxiang |  |  |  |
| 653 | Ziyanfeishuang |  |  |  |
| 656 | Wuhemang |  |  |  |
| 687 | Chunshuangdouxue |  |  |  |
| 693 | Luyiqingqiu |  |  |  |
| 716 | Nijinhongguan |  |  |  |
| 721 | Bingqingyujie |  |  |  |
| 740 | Yulanhe |  |  |  |
| 745 | Zixiao |  |  |  |
| 750 | Posuonufang |  |  |  |
| 753 | Taohualiushui |  |  |  |
| 771 | Rizhaolufeng |  |  |  |
| 774 | Jinjiduli |  |  |  |
| 775 | Zilangfengguang |  |  |  |
| 780 | Fengxian |  |  |  |
| 800 | Nijinhudie |  |  |  |
| 806 | Riluojinshan |  |  |  |
| 813 | Yudie |  |  |  |
| 824 | Qingtangfenxie |  |  |  |
| 834 | Jinjihongling |  |  |  |
| 849 | Zixianchuanzhen |  |  |  |
| 850 | Jinfengdie |  |  |  |
| 851 | Songlinfeibao |  |  |  |
| 865 | Huatianshima |  |  |  |
| 871 | Huihezhanchi |  |  |  |
| 887 | Dahuangmaoju |  |  |  |
| 888 | Jinhongjiaohui |  |  |  |
| 895 | Hongriyinghui |  |  |  |
| 899 | Shouxiantao |  |  |  |
| 939 | Panlongxiqiu |  |  |  |
| 941 | Gangtiecaixian |  |  |  |
| 969 | Panlongchanyun |  |  |  |
| 972 | Lvyitianshi |  |  |  |
| 977 | Fengouhuan |  |  |  |
| 987 | Baoshuiliubing |  |  |  |
| 995 | Quanxiangliuqiao |  |  |  |
| 1018 | Huanshuifugui |  |  |  |
| 1021 | Jincaizhaoxia |  |  |  |
| 1026 | Zhushakuilong |  |  |  |
| 1044 | Hongloumeng |  |  |  |
| 1048 | Xiyangpu |  |  |  |
| 1051 | Jinpantuogui |  |  |  |
| 1052 | Xiuzi |  |  |  |
| 1059 | Jinshixiqiu |  |  |  |
| 1065 | Kaizhaohonglian |  |  |  |
| 1076 | Nihonggongzhu |  |  |  |
| 1099 | Huanglongzhao |  |  |  |
| 1103 | Huangjinpan |  |  |  |
| 1107 | Zifengmingzhu |  |  |  |
| 1122 | Jinfenghuang |  |  |  |
| 1132 | Yujiale |  |  |  |
| 1182 | Tangyuqiushi |  |  |  |
| 1207 | Xueguanchuihua |  |  |  |
| 1210 | Panlongjinwu |  |  |  |

# Supplementary Table Ⅲ

| Table S3.Chrysanthemum cultivar petal type and flower type coding | | | | |
| --- | --- | --- | --- | --- |
| Petal type | Code | Flower type | Code |  |
| flat | 1 | single | 1 |  |
| spoon | 2 | lotus-like | 2 |  |
| tubular | 3 | peony-like | 3 |  |
| anemome | 4 | flat-pan | 4 |  |
| peculiar | 5 | reflexed | 5 |  |
| polytype | 6 | stacked-spherical | 6 |  |
|  |  | spoon-lotus | 7 |  |
|  |  | sparrow-tongue-like | 8 |  |
|  |  | beehive-like | 9 |  |
|  |  | lotus-set-like | 10 |  |
|  |  | scattered-reflexed | 11 |  |
|  |  | spherical-spoon | 12 |  |
|  |  | single-tubiform | 13 |  |
|  |  | straight-tubiform | 14 |  |
|  |  | tubiform-pan | 15 |  |
|  |  | acirular | 16 |  |
|  |  | scattered-tubiform | 17 |  |
|  |  | tubiform-spherical | 18 |  |
|  |  | filiform | 19 |  |
|  |  | fluttered | 20 |  |
|  |  | upswept-bead | 21 |  |
|  |  | jade-like | 22 |  |
|  |  | pendant-bead | 23 |  |
|  |  | flat-anemone | 24 |  |
|  |  | spoon-anemone | 25 |  |
|  |  | tubiform-anemone | 26 |  |
|  |  | whole-anemone | 27 |  |
|  |  | dragon-claw-like | 28 |  |
|  |  | aristata | 29 |  |
|  |  | chenille-like | 30 |  |
